# Reverse transcription of plasma-derived HIV-1 RNA generates multiple artifacts through tRNA(Lys-3)-priming

**DOI:** 10.1128/spectrum.03872-23

**Published:** 2024-03-05

**Authors:** Jarryt Hardy, Els Demecheleer, Marlies Schauvliege, Delfien Staelens, Virginie Mortier, Chris Verhofstede

**Affiliations:** 1 Aids Reference Laboratory, Department of Diagnostic Sciences, Ghent University, Ghent, Belgium; Kumamoto Daigaku, Kumamoto, Japan

**Keywords:** HIV-1, reverse transcription, RNA Sequencing

## Abstract

*In vitro* reverse transcription of full-length HIV-1 RNA extracted from the blood plasma of people living with HIV-1 remains challenging. Here, we describe the initiation of reverse transcription of plasma-derived viral RNA in the absence of an exogenous primer. Real-time PCR and Sanger sequencing were applied to identify the source and to monitor the outcome of this reaction. Results demonstrated that during purification of viral RNA from plasma, tRNA(Lys-3) is co-extracted in a complex with the viral RNA. In the presence of a reverse transcription enzyme, this tRNA(Lys-3) can induce reverse transcription, a reaction that is not confined to transcription of the 5’ end of the viral RNA. A range of cDNA products is generated, most of them indicative for the occurrence of *in vitro* strand transfer events that involve translocation of cDNA from the 5’ end to random positions on the viral RNA. This process results in the formation of cDNAs with large internal deletions. However, near full-length cDNA and cDNA with sequence patterns resembling multiple spliced HIV-1 RNA were also detected. Despite its potential to introduce significant bias in the interpretation of results across various applications, tRNA(Lys-3)-driven reverse transcription has been overlooked thus far. A more in-depth study of this tRNA-driven *in vitro* reaction may provide new insight into the complex process of *in vivo* HIV-1 replication.

**IMPORTANCE:**

The use of silica-based extraction methods for purifying HIV-1 RNA from viral particles is a common practice, but it involves co-extraction of human tRNA(Lys-3) due to the strong interactions between these molecules. This co-extraction becomes particularly significant when the extracted RNA is used in reverse transcription reactions, as the tRNA(Lys-3) then serves as a primer. Reverse transcription from tRNA(Lys-3) is not confined to cDNA synthesis of the 5’ end of the viral RNA but extends across various regions of the viral genome through *in vitro* strand transfer events. Co-extraction of tRNA(Lys-3) has been overlooked thus far, despite its potential to introduce bias in downstream, reverse transcription-related applications. The observed events in the tRNA(Lys-3)-induced *in vitro* reverse transcription resemble *in vivo* replication processes. Therefore, these reactions may offer a unique model to better understand the replication dynamics of HIV-1.

## INTRODUCTION

A prominent feature of the human immunodeficiency virus type 1 (HIV-1) and retroviruses in general is the capacity to reverse transcribe their RNA genome (vRNA) into double-stranded DNA (vDNA) and to integrate this vDNA into the genome of the host. Reverse transcription initiates at the primer binding site (PBS), located just downstream of the 5’ long terminal repeat (LTR) of the vRNA (1). Essential for HIV-1 reverse transcription *in vivo* is the presence of human tRNA(Lys-3). tRNA(Lys-3) includes an 18-nucleotide long anti-PBS sequence that allows base pairing with the complementary PBS on the vRNA (2, 3). Hybridization of tRNA(Lys-3) onto the PBS is facilitated by viral nucleocapsid (NC) proteins. NC proteins help to unfold conformational barriers within the tRNA(Lys-3) molecule, allowing it to bind adequately to the vRNA. Moreover, NC proteins destabilize secondary structures present in the 5’ untranslated region of the vRNA as well (4–6). While other interactions between tRNA(Lys-3) and vRNA may promote the initiation of reverse transcription, their precise molecular specificities are not well understood (3, 7–10). Once tRNA(Lys-3) is bound, the viral reverse transcriptase (RT) can initiate reverse transcription by synthesizing the strong-stop minus-strand cDNA, a short cDNA intermediate that encompasses the U5 and R regions of the 5’ LTR. During minus-strand synthesis, the vRNA is cleaved by the RNase H activity of the RT. Cleavage allows the release of single-stranded cDNA, which can then transfer to a homologous sequence at the 3’ end of the vRNA (11). Following first strand transfer, the RT continues its elongation process to generate a complete LTR sequence, encompassing the U3, R, and U5 regions, before it proceeds to transcribe the coding regions of the HIV genome. While synthesis of minus-strand cDNA is ongoing, plus-strand DNA synthesis initiates. Two polypurine tract sequences in the vRNA that are resistant to the RNAse H activity function as primers for plus-strand DNA synthesis. A second strand transfer is required to complete full-length double-stranded viral DNA (vDNA) with identical LTRs at the 5’ and 3’ terminal ends (1, 12). Besides playing an essential role in the initiation of reverse transcription, the viral NC proteins also facilitate both strand transfers (13, 14). NC proteins are believed to accelerate the rate at which the complementary R sequences anneal. The zinc finger motifs of the NC proteins destabilize the complementary hairpins on the minus-strand cDNA and vRNA, and subsequently, the basic amino acids of the NC proteins support the aggregation of both molecules (15, 16).

Completion of reverse transcription precedes integration of the vDNA into the genome of infected cells. Cells that harbor integrated HIV-1 DNA can persist during antiretroviral therapy (ART) and are commonly referred to as the (pro)viral reservoir (17–20). It is generally assumed that more than 90% of the proviral DNA is replication-defective due to internal deletions, hypermutations, and/or premature stop codons (21, 22). Internal deletions are believed to result from sequence homology-independent template switches during the synthesis of minus-strand cDNA (23). The reverse transcription complex frequently switches during reverse transcription; sequence homology-dependent template switches are even considered essential for the generation of full-length vDNA (24, 25).

Generating full-length vDNA *in vitro* from plasma-derived vRNA is challenging, despite the availability of enzymes capable of transcribing RNAs that exceed the length of the HIV-1 vRNA. Both the presence of stable secondary structures, such as RNA hairpins, and the presence of homopolymers, may facilitate the dissociation of the reverse transcription complex from the RNA template (26, 27). The research described here started as an attempt to optimize reverse transcription of near full-length vRNA, obtained from blood plasma of people living with HIV (PLWH). Preliminary data showed the unexpected presence of HIV-1 cDNA in control reverse transcription reactions to which no exogenous primer was added. Subsequent experiments revealed that the presence of these cDNAs resulted from reverse transcription initiated by co-extracted human tRNA(Lys-3). The interaction between tRNA(Lys-3) and vRNA appears to be remarkably robust, withstanding the denaturing conditions during nucleic acid purification as well as heating at 65°C. Furthermore, short cDNA products that result from the tRNA(Lys-3)-priming can transfer to other positions on the vRNA to proceed elongation beyond the 5’ end of vRNA. These strand transfer events predominantly result in the formation of cDNAs with large internal deletions.

## RESULTS

### *In vitro* reverse transcription of plasma-derived HIV-1 RNA

HIV-1 RNA extracted from plasma samples of participants 09, 30, and 63 was reverse transcribed with SuperScript IV (SSIV; Thermo Fisher Scientific). Information on the study participants is listed in Table S1. Either random hexamers (RH6) or anchored oligo(dT)20 (odT20) primers were used to initiate the reverse transcription reaction. Control reactions that lack the addition of an exogenous primer (no-primer; NP) or reverse transcription enzyme (no-enzyme; NE) were included. To assess to what extent reverse transcription reached the central region or 5’ end of the HIV-1 genome, two real-time (q)PCR assays were performed. One reaction addressed a region in the integrase (INT) coding region of the vRNA (INT qPCR), while the other targeted the 5’ end of the vRNA (LTR qPCR).

**Fig 1:**
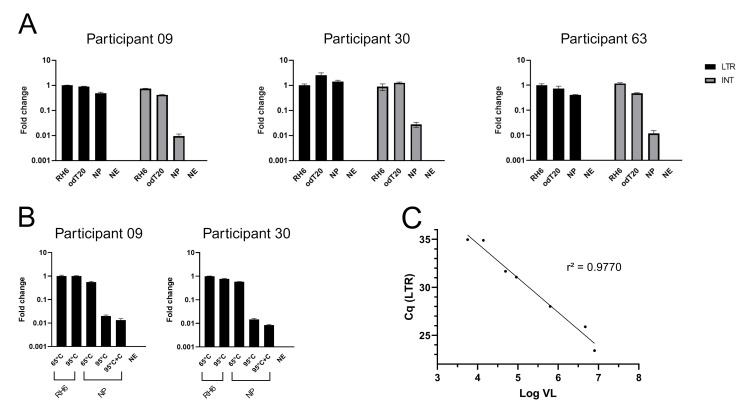
Results of the qPCR performed on reaction products from reverse transcriptions conducted under different conditions. (**A**) LTR (black) and INT (gray) qPCR results for cDNA products obtained after reverse transcription with random hexamers and anchored oligo(dT)20 and in the absence of an exogenous primer (NP). The vRNA used was extracted from the plasma of participants 09, 30, and 63. (**B**) LTR qPCR results for cDNA products obtained after reverse transcription of vRNA preheated at 65°C or at 95°C. The reverse transcription was performed in the presence or absence of a competitor primer (+**C**) intended to block the tRNA-binding. The vRNA used was extracted from the plasma of participants 09 and 30. (**C**) Linear regression analysis to determine the relationship between the viral load (VL, in logarithmic scale) and the amount of LTR cDNA that results from tRNA(Lys-3)-primed reverse transcription (expressed as Cq of the LTR qPCR). Dots represent the results for participants 09, 30, 32, 34 ,42, 63, and 82. Reverse transcription was performed with SuperScript IV RT. Results in (**A**) and (**B**) are expressed as fold changes in Cq values relative to the LTR Cq values obtained after reverse transcription with RH6. NE, control reaction that lacked enzyme.

Reverse transcription with RH6 resulted in highly similar LTR and INT qPCR results, suggesting transcription of both regions with equal efficiency (Fig. 1A; Table S2). Since reverse transcription with odT20 is initiated at the 3’ end and the efficiency of the reverse transcription is supposed to decrease as the extension progresses, it was assumed that only part of the transcripts would reach the 5’ end of the vRNA. Although this assumption should result in lower concentrations of cDNA containing LTR compared to cDNA containing INT, this was not observed. Furthermore, LTR quantification cycle (Cq) values for the NP reverse transcription did not significantly differ from those obtained for products of reverse transcription reactions with RH6 or odT20, which suggested the initiation of reverse transcription of the 5’ LTR in the absence of an exogenous primer. Results for the INT qPCR performed on the products of NP reactions, on the other hand, were much lower than those obtained for reactions with RH6 and odT20, but still above the threshold (Fig. 1A). No fluorescent signal above the threshold was observed for reactions run without enzyme (NE) and for reactions run with RNA of HIV-1-negative participants. This excluded the presence of HIV DNA in the RNA extracts, as well as the presence of other sources of PCR contamination.

A linear regression analysis determined the relationship between the plasma viral load and the Cq values resulting from LTR qPCR after NP reverse transcription. Reverse transcription was carried out for the samples of seven participants with diverse viral loads (participants 09, 30, 32, 34, 42, 63, and 82; Table S1). The results of this analysis clearly revealed a correlation between the amount of cDNA products generated in the absence of an exogenous primer and the vRNA input (Fig. 1C). Considering the consecutive dilutions over the different steps of the procedure, the amount of vRNA introduced to the final qPCR reaction for the plasma sample with the lowest viral load (3.78 log_10_ copies/mL; participant 32) corresponded to a calculated equivalent of 17 vRNA molecules.

**Fig 2:**
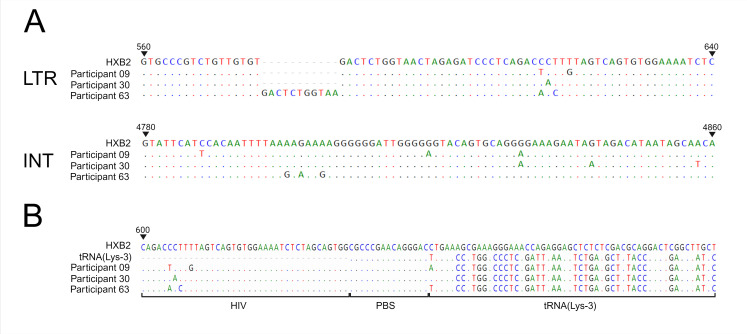
Nucleotide sequences of (**A**) amplicons generated through LTR and INT nested PCR for cDNA products obtained from NP reverse transcription and (**B**) amplicons generated through a nested PCR designed to amplify the HIV-1/tRNA(Lys-3) hybrid molecules that resulted from two consecutive reverse transcription reactions. The vRNA used was extracted from the plasma of participants 09, 30, and 63. The sequences are aligned to the HXB2 HIV-1 reference genome.

Nested PCRs were performed to further confirm the presence of LTR- and INT-specific cDNA after NP reverse transcription. Agarose gel electrophoresis showed the presence of amplicons for both reactions (Fig. S2 and S3). Sequence analysis confirmed that these amplicons represented participant-specific LTR and INT sequences (Fig. 2A).

In an effort to determine to what extent the observed reaction was RT-dependent, NP reverse transcription was performed using four different RTs. Enzymes were selected based on their intrinsic properties, with a particular attention for the functionality of the RNase H domain. MMuLV (New England Biolabs), SSIV (Thermo Fisher Scientific), and PrimeScript II (PSII; Takara Bio) are moloney murine leukemia virus (MMLV) RT mutants; Transcriptor (TRT; Roche Life Science) is a modified avian myeloblastosis virus (AMV) RT. MMuLV and TRT possess a functional RNase H domain, while SSIV has been manipulated to reduce the RNase H activity and PSII has a non-functional RNase H domain. No substantial differences were observed in the outcome of these RT reactions, suggesting that neither the origin of the enzyme nor the RNase H activity affects cDNA synthesis in the absence of an exogenous primer (Fig. S4).

### cDNA synthesis in NP reactions is initiated by tRNA(Lys-3)

Considering the detection of substantial amounts of cDNA of the 5’ end of the vRNA in NP reactions, we considered the potential priming by human tRNA(Lys-3). cDNA products from NP reverse transcription were subjected to an additional reverse transcription that included a sense primer located in the R region of the HIV-1 genome. Relying consecutively on the DNA-dependent and RNA-dependent polymerase activity of the RT (TRT), synthesis of a DNA copy of HIV-1 cDNA linked to human tRNA(Lys-3) was anticipated. Nested PCR, with sense primers complementary to the 5’LTR of the vRNA and antisense primers complementary to tRNA(Lys-3), allowed amplification and sequencing of the resulting amplicon. The results confirmed the presence of amplicons with participant-specific HIV-1 sequences linked to human tRNA(Lys-3) (Fig. 2B).

To check whether the tRNA(Lys-3)-vRNA interaction and priming can be disrupted by heat treatment, vRNA from participants 09 and 30 was heated at 95°C prior to reverse transcription. Heat treatment resulted in a substantial reduction in LTR qPCR results, both for reverse transcription with SSIV (Fig. 1B; Table S3) and TRT (Fig. S5; Table S3). Heat treatment, however, did not nullify the LTR signal and thus was not able to fully prevent tRNA(Lys-3)-priming. Heat treatment of the vRNA at 95°C for 5 minutes did not substantially alter the outcome of the reverse transcription with RH6, ruling out extensive degradation of the vRNA at 95°C (Table S3). Subsequently, we assessed the effect of heating in combination with the addition of a primer with a sequence identical to the HIV-1 PBS (competitor primer, Table S4). This primer was expected to compete with the vRNA for tRNA-binding but its presence only mildly influenced the LTR qPCR result (Table S3).

### Strand transfer during tRNA-primed reverse transcription

The results described above provided solid evidence for the occurrence of tRNA(Lys-3)-primed reverse transcription, elucidating the observed high concentration of cDNA of the 5’ end of vRNA after reverse transcription without addition of an exogenous primer. However, low concentrations of cDNA containing integrase sequences were also detected among the reverse transcription products (Fig. 1A). Therefore, we hypothesized the occurrence of *in vitro* strand transfer events during tRNA-driven reverse transcription. To consider this hypothesis, we checked for the presence of cDNA products that include the complete LTR sequence. A complete LTR sequence, encompassing the U3, R, and U5 regions, can only be present if strand transfer has occurred. In order to obtain sufficient amounts of HIV-1 cDNA for these experiments, vRNA was extracted from a plasma sample with high viral load (participant 25). A nested PCR with sense primers in U3 and antisense primers in U5 was performed to demonstrate the presence of U3-R-U5 cDNA after NP reverse transcription. Gel electrophoresis and sequence analysis confirmed the presence of complete LTR sequences (Fig. S6).

**Fig 3:**
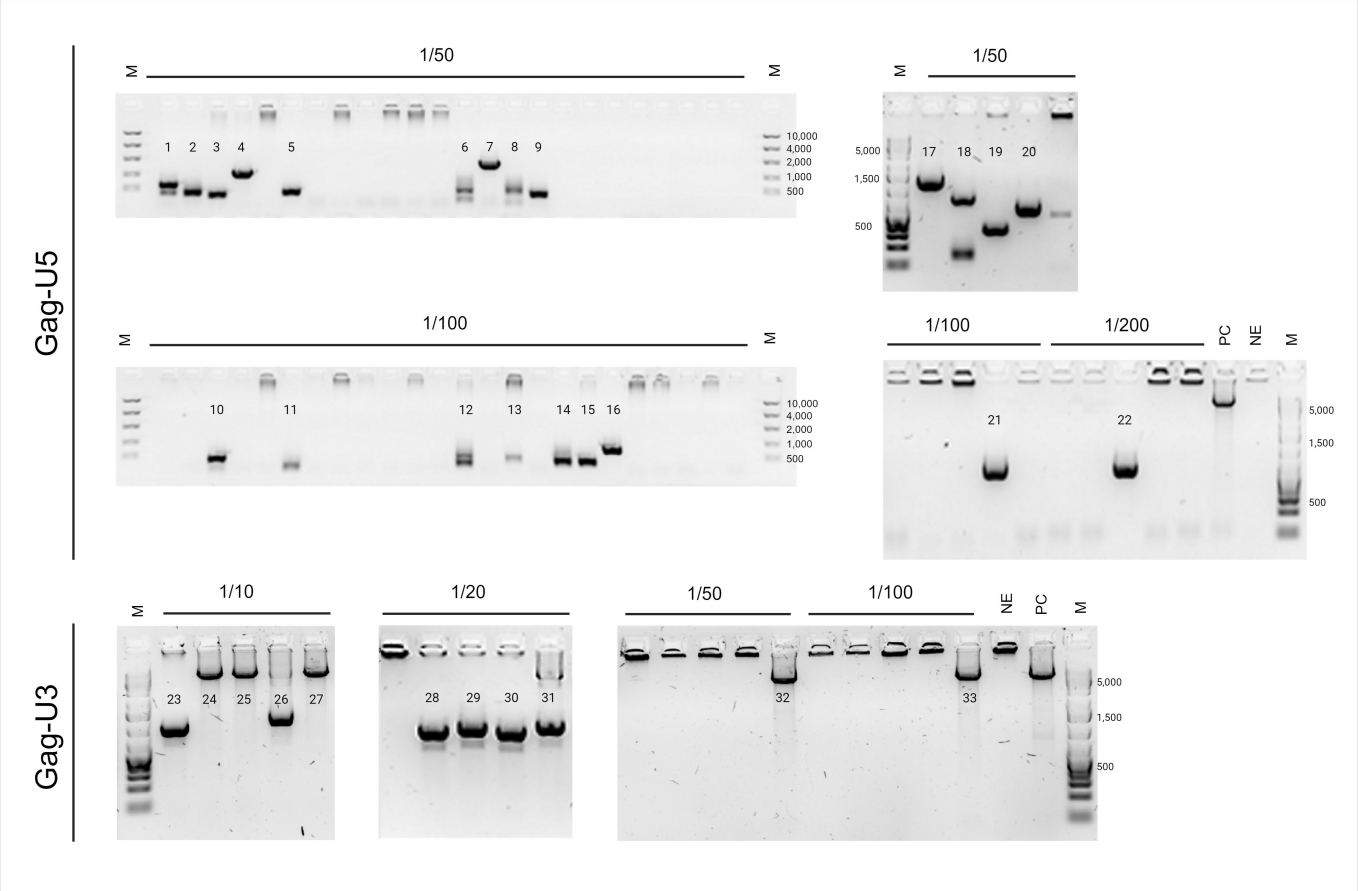
Agarose gels showing the amplicons generated through Gag-U5 and Gag-U3 nested PCRs, cDNA was obtained from NP reverse transcription. cDNA products were diluted 1/10, 1/20, 1/50, 1/100, and 1/200, respectively. Replicate nested PCR reactions were run for each dilution. vRNA was extracted from the plasma of participant 25 and reverse transcription was performed with SuperScript IV RT. Amplicons that were considered eligible for sequencing were numbered and the same numeration was used to identify the corresponding sequence (Fig. 4). NE, no enzyme control; PC, positive control (8E5 DNA); M, molecular weight marker.

Additional nested PCRs were then performed to determine to what extent longer cDNA fragments, reaching beyond the LTR, were generated. A nested PCR with sense primers located downstream of the PBS, in the Gag gene, and antisense primers located in U5 (Gag-U5 PCR) and a nested PCR with sense primers in Gag and antisense primers in U3 (Gag-U3 PCR) were performed. For these nested PCR reactions, cDNA from NP reactions was diluted and replicate reactions were performed for each dilution to increase the likelihood of amplifying individual cDNAs. Agarose gel electrophoresis revealed amplicons that varied in length, ranging from several hundreds to about 2,000 nucleotides, in both the Gag-U5 PCR and the Gag-U3 PCR (Fig. 3).

**Fig 4:**
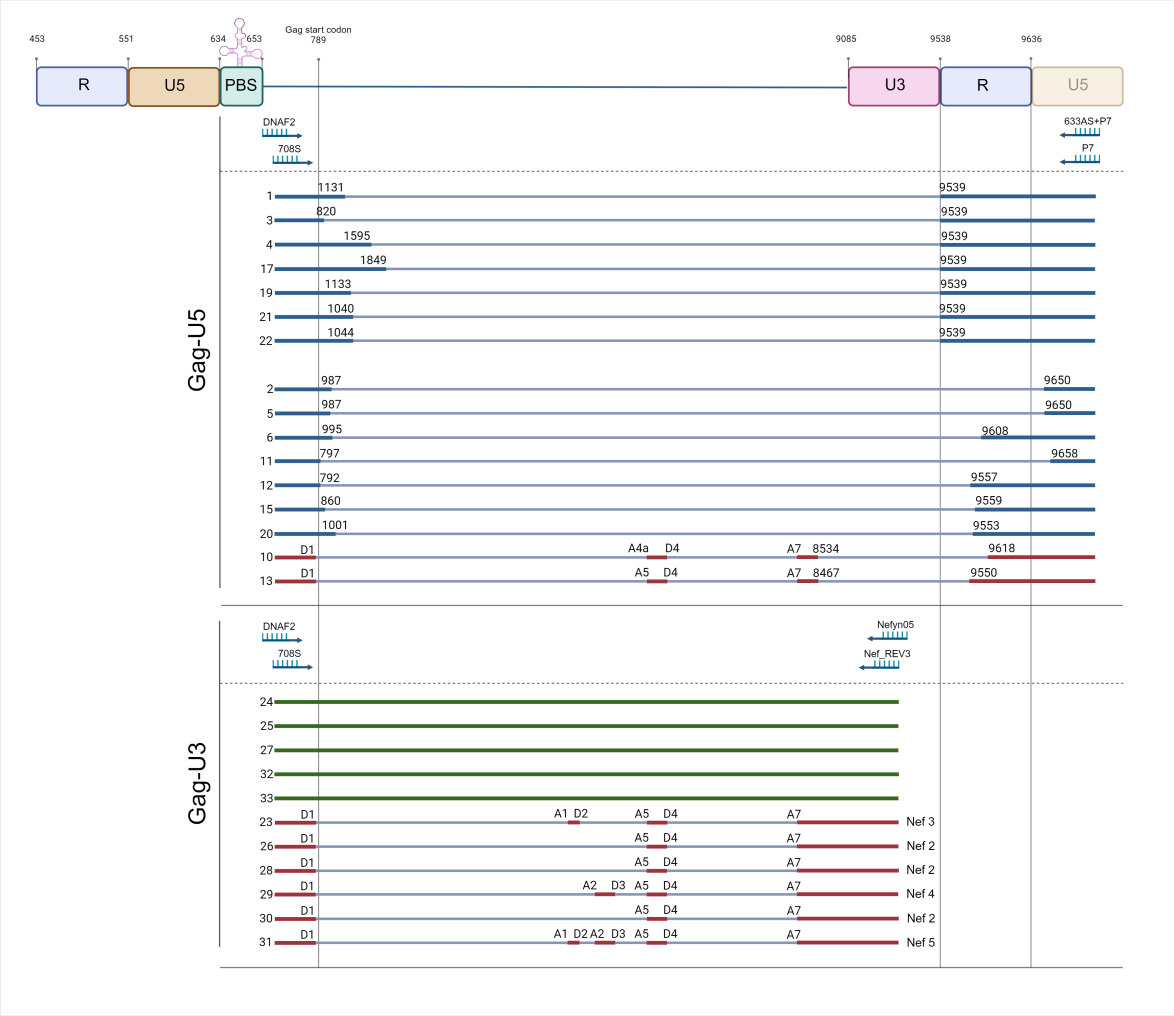
Schematic representation of the sequences of amplicons generated through Gag-U5 and Gag-U3 nested PCR (see Fig. 3). The primers used in the nested PCR are depicted above. Amplicons are color-coded: blue, amplicons with a large internal deletion; green, intact, near-full length amplicons; and red, amplicons with breakpoints that correspond to multiple spliced HIV-1 RNA donor (**D**) and acceptor (**A**) splice sites. Amplicons 7, 8, 9, 14, 16, and 18 were not included because of failed sequence reactions.

Sequence analysis of the Gag-U5 amplicons showed patterns indicative of two potential strand transfer mechanisms (Fig. 4). The sequences of amplicons 1, 3, 4, 17, 19, 21, and 22 suggested completion of strong-stop minus-strand cDNA synthesis followed by translocation of the cDNA to a random position on the vRNA. These homology-independent strand transfers resulted in cDNA transcripts with large internal deletions. The 3’ breakpoints of these deletions coincided with the homopolymer G triplets that define the 5’ end of R. The 5’ breakpoints varied between amplicons but were all located in Gag. The sequences of amplicons 2, 5, 6, 11, 12, 15, and 20 (Fig. 4) suggested transfer of cDNA before reverse transcription reached the 5’ end of the vRNA. These homology-independent strand transfers also resulted in cDNA with 5’ breakpoints located in the Gag gene but the 3´ breakpoints were located in R or U5. Amplicons 7 and 10 displayed more complex sequence patterns that reflected strand transfer of prematurely terminated cDNA to multiple spliced HIV-1 messenger (ms)RNA molecules; more specifically, a sequence matching the msRNA coding for Rev (amplicon 7) and a sequence matching the msRNA coding for Nef (amplicon 10), were detected. cDNA copies of msRNA were observed even more frequently when running the Gag-U3 nested PCR. Amplicons 23, 26, 28, 29, 30, and 31 (Fig. 4) contained sequence fragments of different Nef msRNA transcripts. Moreover, the Gag-U3 nested PCR also resulted in the generation of amplicons with intact HIV-1 sequences (amplicons 24, 25, 27, 32, and 33, Fig. 4). In contrast to the Gag-U5 PCR, none of the amplicons generated in the Gag-U3 PCR showed evidence of sequence homology-independent strand transfers.

## DISCUSSION

We intended to optimize the conditions for full-length HIV-1 RNA reverse transcription, which led to the observation that human tRNA(Lys-3) molecules are co-extracted in complex with vRNA from the plasma of PLWH and can function as primer for reverse transcription. During HIV-1 assembly, two genomic RNA molecules are packaged into the viral particle, along with various noncoding host RNAs, including tRNA(Lys-3). It has been estimated that approximately 20 tRNA(Lys) molecules are packaged into each viral particle, around 8 of them being tRNA(Lys-3) (28–30). Hybridization of tRNA(Lys-3) on the PBS sequence of the vRNA is required for viral replication but is not supposed to occur spontaneously at physiological temperatures, as both the PBS and tRNA(Lys-3) have highly structured configurations (5). Our observations led to the conclusion that tRNA(Lys-3) is already bound to the vRNA in the viral particles and that this interaction is particularly rigid. Because of their small size, free tRNA(Lys-3) molecules are not expected to bind to the silica membrane that is used in the QIAamp viral RNA extraction kit. Therefore, their presence in the RNA extract can only be resolved by the extraction of tRNA(Lys-3) in complex with vRNA. The interactions between both molecules must be very rigid as the denaturing conditions during the extraction procedure are supposed to disrupt all hydrogen bonds. Heating the vRNA at 95°C before reverse transcription substantially reduced the tRNA(Lys-3)-induced reverse transcription but did not nullify it.

One other report referred to tRNA-primed *in vitro* reverse transcription and co-extraction of vRNA and tRNA (31). This study, however, relied on RNA purification from viral particles produced in a transfected cell line. It is important to note that, in this work, a guanidinium isothiocyanate procedure was used for RNA extraction, demonstrating that the interaction between tRNA and vRNA also withstands the denaturing conditions of this commonly used extraction method. Spontaneous reverse transcription of HIV-1 RNA in the absence of an exogenous primer has, to our knowledge, not been reported before. The presence of tRNA-priming may elucidate the high sensitivity of commercial HIV-1 viral load assays that target the 5’ LTR region. tRNA(Lys3)-priming will allow the synthesis of LTR cDNA regardless of the binding capacity of the exogenous primer, significantly enhancing the sensitivity of these assays.

Spontaneous priming and initiation of reverse transcription has been documented before for other RNA viruses, including hepatitis C, enteroviruses, and dengue virus. In all these cases, secondary structures of the vRNA have been considered as initiators of the reverse transcription, though without firm proof (32–34). Although our study clearly demonstrated the role of tRNA(Lys-3) in the spontaneous generation of HIV-1 cDNA during *in vitro* reverse transcription, we cannot entirely rule out a potential additional effect of priming caused by secondary structures.

tRNA(Lys-3)-induced reverse transcription was not limited to the formation of DNA copies of the 5’ end of vRNA. Straightforward evidence for the occurrence of homology-dependent as well as homology-independent strand transfer, resulting in the formation of cDNA containing complete LTR sequences and/or internal regions of the viral genome, is provided. Prior research revealed the crucial role of the HIV-1 NC proteins in facilitating successful first strand cDNA transfer (35–37). NC proteins promote the displacement of short RNAs that persist after RNase H cleavage, thereby releasing single-stranded cDNA. NC proteins also have a significance in terms of destabilizing RNA and DNA hairpins. Unfolding these hairpins is essential for the formation of the DNA-RNA heteroduplex in strand transfer (38–40). Furthermore, it is supposed that the close proximity of the two co-packaged RNAs in the viral particles is essential for proper template switching (41, 42). Our research, however, shows that strand transfer can occur in the absence of NC proteins or close proximity of the RNA, and also in absence of RNAse H activity.

A final important observation is that a considerable amount of the cDNAs generated through tRNA-priming represented msRNAs, suggesting the presence of these msRNAs in plasma-derived RNA extracts. Spliced RNA transcripts do have many of the RNA elements fundamental for viral replication, but they lack the stem-loop 3 (SL3) structure. Non-canonical base pairs between nucleotides 306 to 309 and 328 to 331 (UUUU:GGAG motif) in the SL3 are considered to create a high-affinity binding site for NC proteins, directing the selective packaging of genomic RNA in the presence of spliced RNAs (43). Nevertheless, emerging evidence suggests that spliced RNA transcripts can form heterodimers with genomic RNA, allowing them to be packaged into the viral particles (44, 45). Additional research has confirmed that the reverse transcription machinery does not discriminate between genomic and spliced RNA and that spliced RNA transcripts can be reverse transcribed into cDNA (46, 47). The proportion of msRNA relative to genomic RNA in plasma virions, however, remains unclear. A long-range Gag-3´ LTR amplification and sequencing method for vRNA has been recently published (48). From the results of this assay, the authors concluded that only 65% of the plasma HIV-1 RNA is genetically intact. Among the sequences that were not intact, 37% showed deletions exceeding 100 base pairs. Remarkably, 8% of these deleted sequences had deletion breakpoints that coincided with known msRNA splice acceptor and donor sites (48).

Some limitations of our study need to be considered. One notable limitation is the potential introduction of bias in the distribution of cDNA products generated by tRNA(Lys3)-priming due to the choice of PCR primers. Primer selection restricts the amplification of cDNAs to those that encompass the primer positions, which implies that certain cDNAs will be effortlessly detected while others will not be amplified. Another PCR-related concern is the potential preferential amplification of shorter cDNA products, like the ones with large internal deletions or, to a lesser extent, those that result from the reverse transcription of msRNA. This may result in an overestimation of their presence. A recent report showed the length-related reduction in PCR efficiency during the amplification of HIV-1 DNA (49). Therefore, our findings can only be interpreted as evidence for the occurrence of strand transfer during *in vitro* reverse transcription and not to decide on the proportion of the different products or the impact that these strand transfers can have on various downstream applications. They do point out, however, the importance of including a no-primer control reaction to assess the potential repercussions of tRNA(Lys-3)-priming on any reverse transcription reaction using vRNA. Another limitation is that we cannot take into account potential differences in the performance of commercial RTs in comparison with the HIV-1 RT. Commercially available enzymes derive from different retroviral sources and have been manipulated to improve their efficiency. It is important to note that, in comparison with the HIV-1 RT, MMLV- and AMV-recombinants are considered to have a higher fidelity. Furthermore, it has been demonstrated that the processivity of MMLV enzymes is substantially higher than that of the HIV-1 RT (50, 51). Caution is therefore required when our observations are extrapolated to the *in vivo* HIV-1 replication cycle.

In conclusion, conventional nucleic acid purification methods used for extraction of HIV-1 RNA from plasma co-extract tRNA(Lys-3). These tRNA(Lys-3) molecules will act as primers in reverse transcription, which may introduce bias in the interpretation of results. Increasing the temperature to 95°C prior to reverse transcription can reduce the effect of tRNA(Lys-3)-primed reverse transcription but will not completely nullify it. Our findings also highlight the robustness of the interaction between vRNA and tRNA(Lys-3), an interaction that endures denaturing conditions in RNA purification, as well as heating at 65°C. This interaction can present an interesting new target for future antiretroviral drug development.

## MATERIALS AND METHODS

### Study approval and samples

EDTA plasma samples from eight PLWH were selected from the ARL/ARC Biobank (BC-4364). Blood samples from four participants (09, 25, 30, and 63) were collected during the acute phase of infection and contained high viral loads (respectively, 6.67, 8.74, 6.90, and 5.80 log_10_ copies/mL). The remaining four participants (32, 34, 42, and 82) had blood samples collected during the chronic phase of infection and were selected to include a range of lower viral loads (respectively 3.78, 4.17, 4.70, and 4.96 log_10_ copies/mL). Characteristics of the study participants are detailed in Table S1. Samples from two HIV-negative individuals were included for control reactions.

### Viral load

HIV-1 viral load was determined in EDTA blood plasma using the Cobas HIV-1 test (Roche Diagnostic) and was run on the Cobas 4800 system. Linear regression was performed using Prism 9.5.1 software (GraphPad) to determine the relationship between the viral load and the results of the LTR qPCR.

### RNA extraction

HIV-1 RNA was extracted from 140 µL plasma using the QIAamp Viral RNA Mini kit (Qiagen) following the manufacturer’s instructions with elution of the purified RNA in 60 µL of elution buffer. All extractions were carried out in triplicate; the RNA was pooled and either used immediately or stored at −20°C.

### Reverse transcription

Diverse enzymes were used for reverse transcription: PSII (Takara Bio), SSIV (Thermo Fisher Scientific), TRT (Roche Life Science), and MMuLV (New England Biolabs). Reverse transcription reactions were carried out according to the manufacturer’s instructions for each enzyme. A minor modification was introduced for the SSIV and MMuLV enzymes: each reaction contained 20 units (U) of recombinant RNase inhibitor (Takara Bio). Prior to reverse transcription, the vRNA was heated at 65°C for 5 minutes to denature secondary structures and linearize the vRNA. Reverse transcription reactions were performed with 5 µL of vRNA and primed with either 2.5 µM of random hexamers (Invitrogen) or 2.5 µM of anchored oligo(dT)20 (Invitrogen). To facilitate annealing of the RH6, an additional incubation step was carried out in accordance with the manufacturers’ instructions: 10 minutes at 30°C for PSII, 10 minutes at 23°C for SSIV, 10 minutes at 25°C for TRT, and 5 minutes at 25°C for MMuLV. To monitor non-specific reactions, contamination, or the presence of vDNA, control reactions that either lacked primer (NP) or reverse transcription enzyme (NE) were included. All reverse transcription reactions were performed in triplicate.

### Real-time PCR

Real-time PCR was carried out using primers and probes positioned either in the LTR region at the 5´ end of the vRNA (LTR qPCR) or in the integrase gene (INT qPCR). Reactions were performed with the Quantitect Multiplex PCR Kit (Qiagen) and run on the LightCycler 480 (Roche Diagnostics). Primers and hydrolysis probes used for LTR and INT qPCR were adapted from published studies (52, 53). Table S4 lists the primers and probes used. Both the LTR and INT qPCR are routinely used in-house for HIV diagnosis in infants and have been thoroughly validated. Each qPCR reaction was prepared in 50 μL-reaction volumes containing 25 µL of 2× Quantitect multiplex master mix, 0.4 µM of each primer and probe, and 5 µL of the reverse transcription product. Thermocycling conditions were as follows: 2 minutes at 50°C and 10 minutes at 95°C, followed by 50 cycles of 15 seconds at 95°C and 1 minute at 60°C, and a final 3 minutes at 37°C. Fluorescent signal detection started from cycle 6 onward. In each run, a dilution series of DNA extracted from a known number of 8E5 cells (that contain a single HIV-1 DNA copy per cell) was included to construct a standard curve. Negative control reactions with molecular biology grade water, instead of cDNA, were included in each run. Each analysis was performed in triplicate. Results are expressed as fold changes in Cq relative to the LTR Cq values obtained for the reverse transcription reactions with RH6.

### Nested PCR

Reverse transcription products were amplified through nested PCR using the Platinum SuperFi II PCR Master Mix (Thermo Fisher Scientific). The 25 μL reaction mixture for the first amplification reaction contained 12.5 µL of 2× Platinum SuperFi II PCR master mix, 0.25 µM of sense and antisense primers, and 2.5 µL of cDNA. First-round PCR products (1.5 µL) were immediately transferred to a second 25 μL-reaction mixture with Platinum SuperFi II Green PCR Master Mix. Depending on the PCR target, the primers and cycling conditions differed (Table S4). As for the cycling conditions, the manufacturer’s instructions were followed but dependent on the amplicon size the extension time was adapted: 20 seconds for the INT PCR, 30 seconds for the LTR, tRNA, and U3-U5 PCR, and 5 minutes for the Gag-U5 and Gag-U3 PCR. Electrophoresis on a 2% agarose gel was used to visualize positive reactions, followed by purification of the amplicons with the QIAquick PCR Purification kit (Qiagen).

### Sanger sequencing

Sequence analysis was performed with the BigDye Terminator v3.1 Cycle Sequencing kit (Applied Biosystems). Reaction mixtures consisted of 1.75 µL of 5× sequencing buffer, 0.5 µL of BigDye Terminator v3.1 Ready Reaction Mix, 0.5 µM of primer, and 2 µL of purified amplicon. The different primers used for sequencing are listed in Table S4. Cycling conditions were as follows: 30 cycles of 30 seconds at 95°C, 15 seconds at 55°C, and 4 seconds at 60°C, followed by incubation at 4°C. Reaction products were purified with Agencourt CleanSEQ beads (Beckman Coulter) before being transferred to the 3500 Genetic Analyzer (Applied Biosystems). Chromatograms were proofread using Chromas (Technelysium Pty) and manually aligned to the HXB2 reference using the BioEdit sequence alignment editor.

### Detection of HIV-1 cDNA/tRNA(Lys-3) hybrids

In an attempt to substantiate the presence of cDNA/tRNA(Lys-3) hybrid molecules, an additional reverse transcription reaction was performed after the initial tRNA-primed reverse transcription. Both the first and second reactions were performed with TRT. In the second reaction, 5 µL of the product of the first reverse transcription was used as template and 0.5 µM of an HIV-specific sense primer, F1fw1, was added. F1fw1 is positioned in the R region of the HIV-1 LTR and initiates the generation of a plus-strand HIV-1 cDNA, relying on the DNA-dependent polymerase activity of TRT. Subsequently, the enzyme switches to its RNA-dependent activity to reverse transcribe the attached tRNA molecule. DNA copies of the HIV-1 cDNA-tRNA(Lys-3) hybrids were then amplified using a nested PCR with sense primers positioned in the HIV-1 5’LTR and antisense primers for the human tRNA sequence (Table S4).

### Heat-disruption of the interaction between tRNA(Lys-3) and vRNA

In an attempt to disrupt the interaction between tRNA(Lys-3) and vRNA, the vRNA was heated at 95°C for 5 minutes followed by immediate cooling on ice. Subsequently, reverse transcription was performed with either RH6 or without the addition of an external primer. In addition, the effect of heat treatment was evaluated in combination with a primer designed to compete with vRNA for tRNA(Lys-3) binding. The sequence of the competitor primer was identical to the HIV-1 PBS sequence (Table S4).

## Data Availability

The data underlying this article is available both in the article itself and in its online supplementary material.

## SUPPLEMENTAL MATERIAL

Supplemental material spectrum.03872-23-s0001.docx Fig. S1 to S6, Tables S1 to S4.

ASM does not own the copyrights to Supplemental Material that may be linked to, or accessed through, an article. The authors have granted ASM a non-exclusive, world-wide license to publish the Supplemental Material files. Please contact the corresponding author directly for reuse.
