# Supplementary material for: Reverse transcription of plasma-derived HIV-1 RNA generates multiple artifacts through tRNA(Lys-3)-priming
Source: Microbiol Spectr. 2024 Mar 5;12(4):e03872-23. doi: 10.1128/spectrum.03872-23 (PMC10986323; doi:10.1128/spectrum.03872-23)
Supplement: Supplemental material — Fig. S1 to S6, Tables S1 to S4. [file spectrum.03872-23-s0001.docx]

**Supplemental material**

**
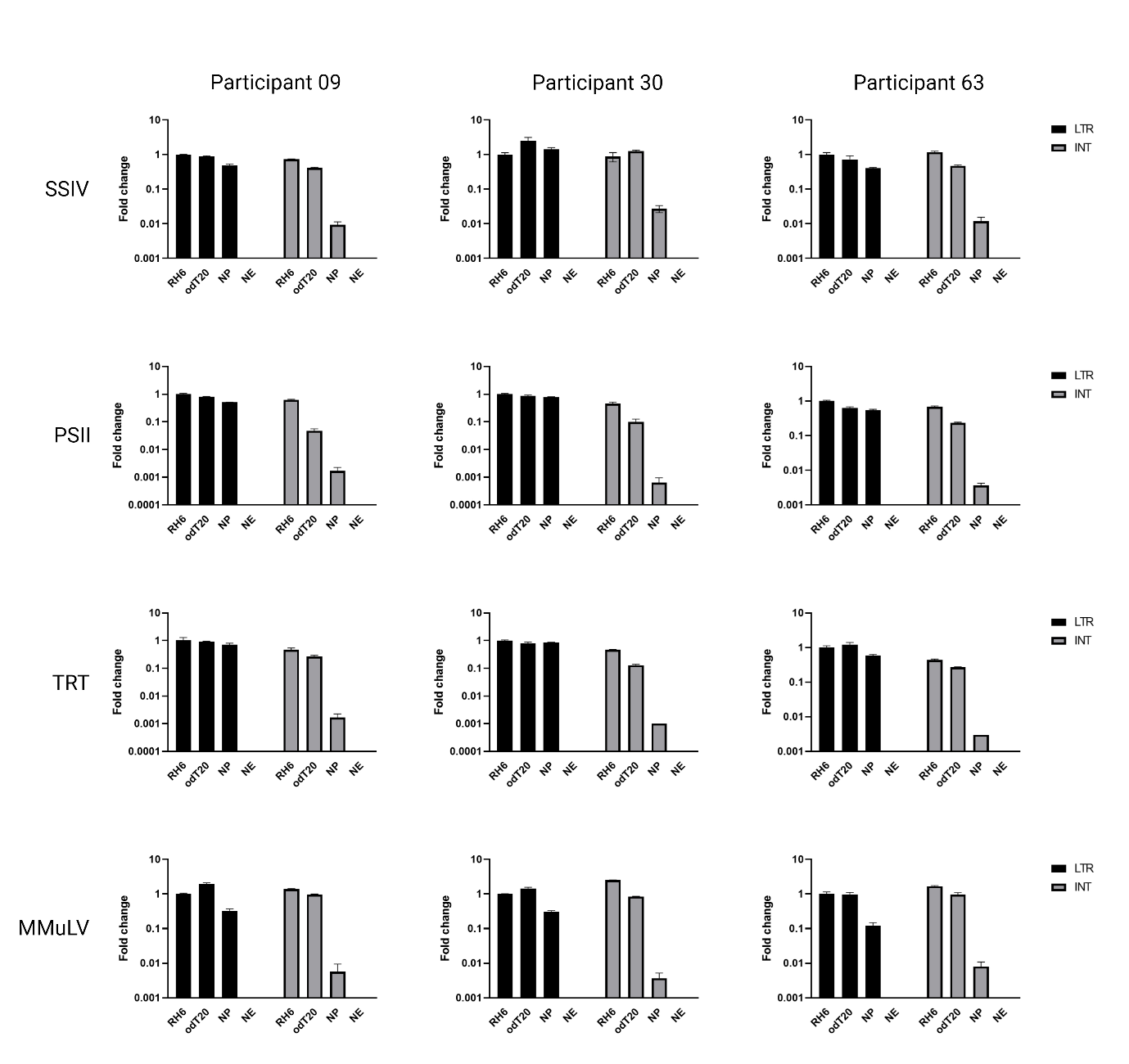
**

**Fig. S1** Results of the LTR (black) and INT (grey) qPCR for cDNA products obtained after reverse transcription with different reverse transcriptases: SuperScript IV (SSIV), PrimeScript II (PSII), Transcriptor (TRT) and MMuLV. Reverse transcription was initiated using random hexamers (RH6), anchored oligo(dT)20 (odT20), or in absence of primer (NP). The vRNA used was extracted from the plasma of participants 09, 30 and 63. Results are expressed as fold changes in Cq values relative to the Cq values obtained from the reactions performed with RH6. NE; no enzyme control.


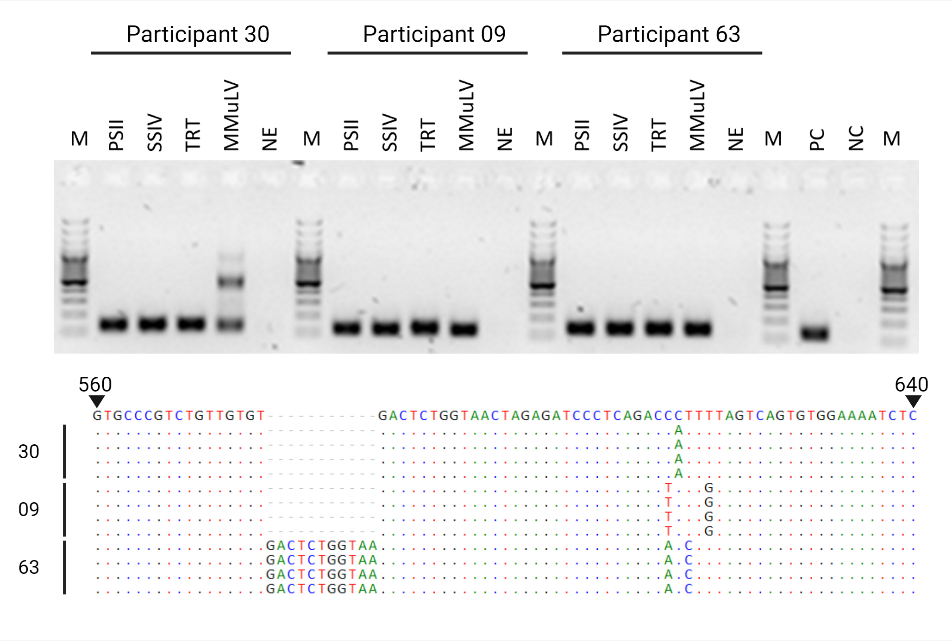


**Fig. S2** Agarose gel of the amplicons generated through nested LTR PCR with their corresponding sequences. Reverse transcription was performed with different reverse transcriptases: SuperScript IV (SSIV), PrimeScript II (PSII), Transcriptor (TRT) and MMuLV, in absence of an exogenous primer. The vRNA used was extracted from the plasma of participants 30, 09 and 63. NE; no enzyme control, PC; positive control (8E5 DNA), NC; negative control (molecular biology grade water), M; molecular weight marker.


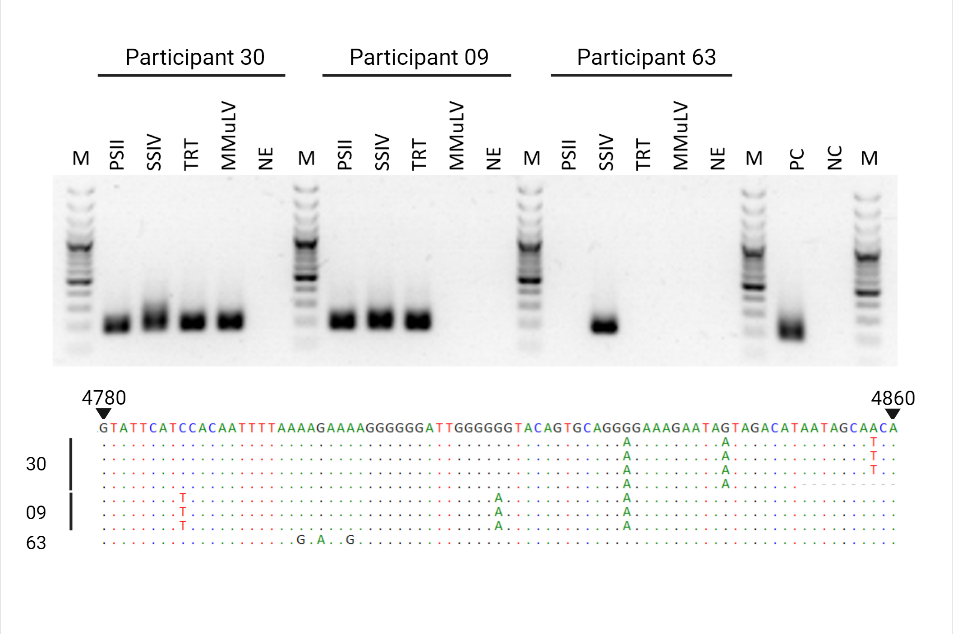


**Fig. S3** Agarose gel of the amplicons generated through nested INT PCR with their corresponding sequences. Reverse transcription was performed with different reverse transcriptases: SuperScript IV (SSIV), PrimeScript II (PSII), Transcriptor (TRT) and MMuLV, in absence of an exogenous primer. The vRNA used was extracted from the plasma of participants 30, 09 and 63. NE; no enzyme control, PC; positive control (8E5 DNA), NC; negative control (molecular biology grade water), M; molecular weight marker.


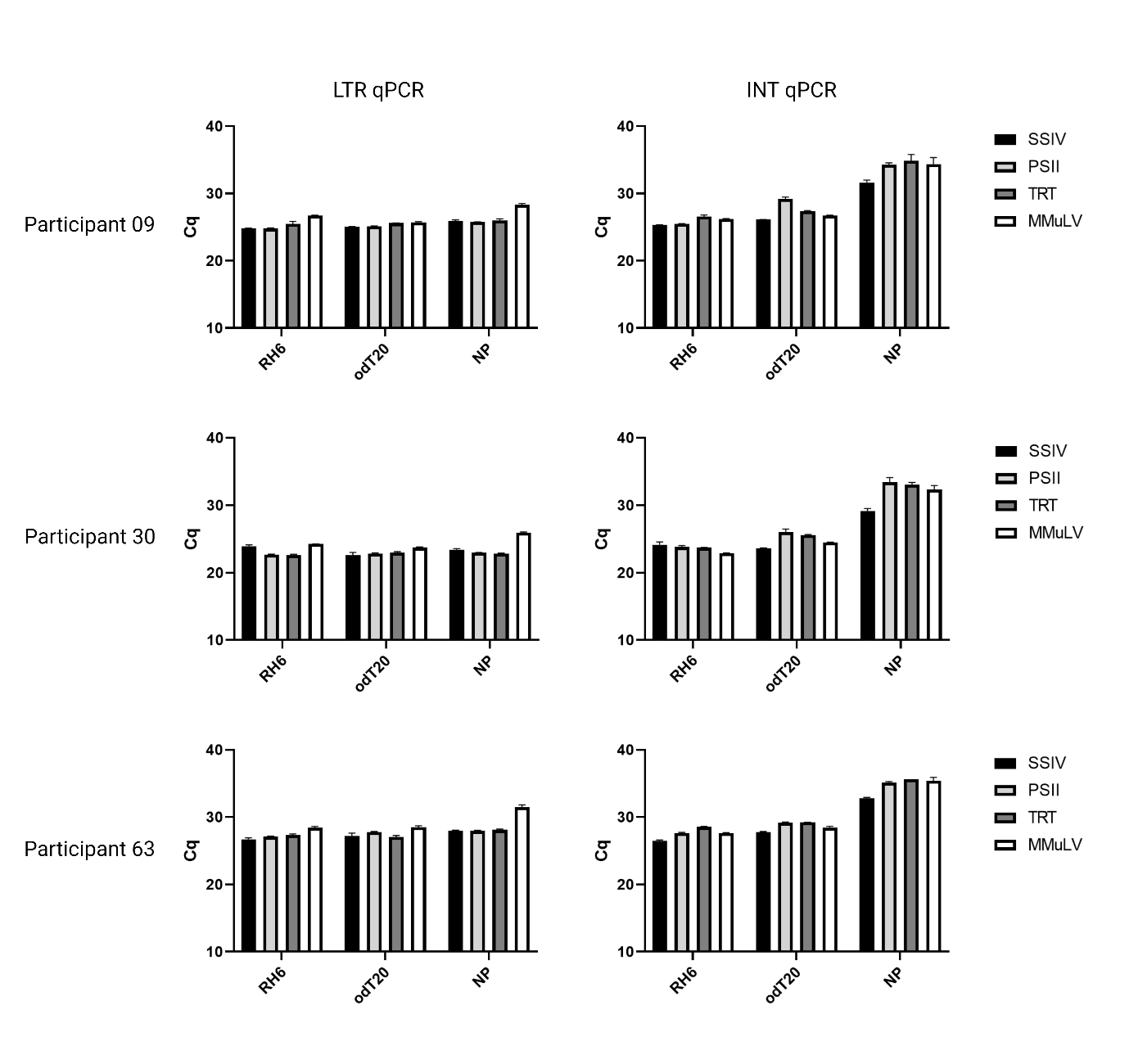


**Fig. S4** Results of the LTR (black) qPCR and INT (grey) qPCR for cDNA products from reverse transcription reactions conducted with different reverse transcriptases: SuperScript IV (SSIV; black), PrimeScript II (PSII; light grey), Transcriptor (TRT; dark grey) and MMuLV (white). Reverse transcription was initiated using random hexamers (RH6), anchored oligodT20 (odT20), or in the absence of an exogenous primer (NP). The vRNA used was extracted from the plasma of participants 09, 30 and 63.


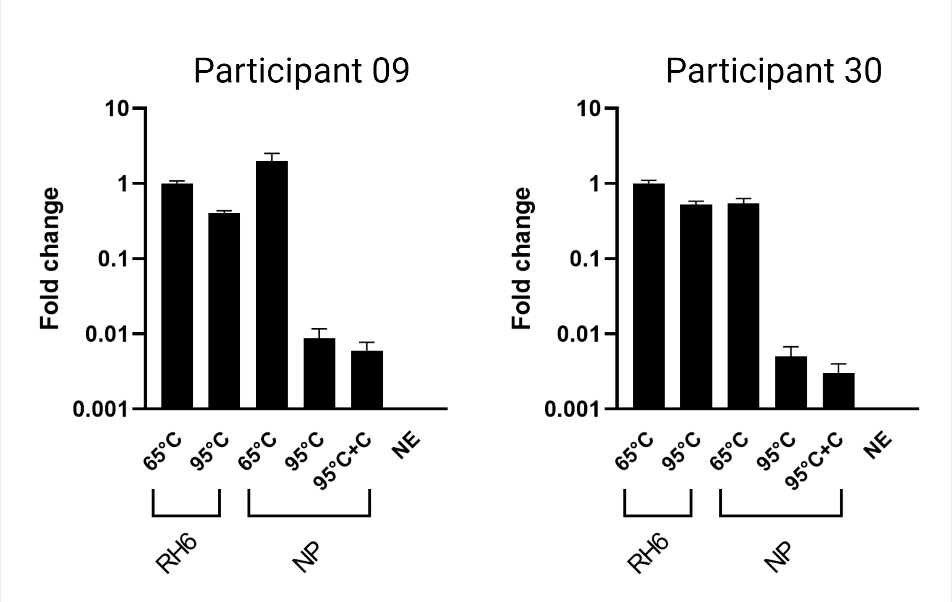


**Fig. S5** LTR qPCR results for cDNA generated after preheating the vRNA at 65 °C and at 95 °C, and with (+C) or without addition of a primer that was designed to compete with vRNA for tRNA(Lys-3)-binding. Reverse transcription was performed with Transcriptor (TRT) and initiated with RH6, or run in absence of an exogenous primer (NP). The vRNA used was extracted from the plasma of participants 09 and 30. NE; no enzyme control.


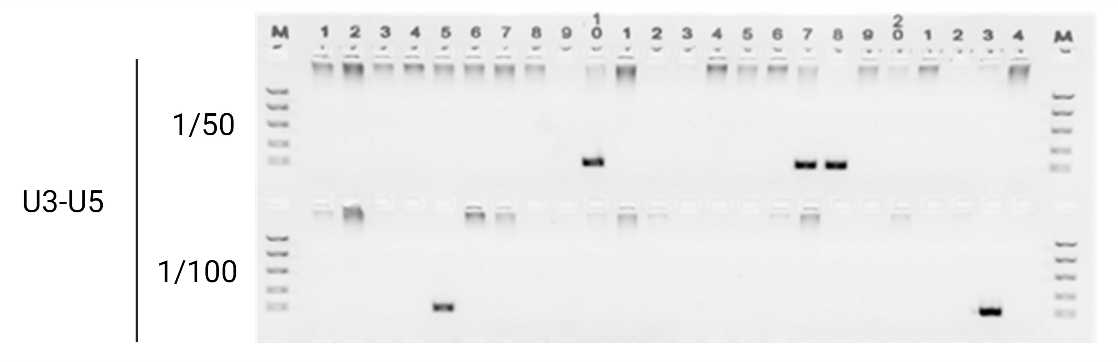


**Fig. S6** Amplicons obtained through the U3-U5 nested PCR on cDNA obtained from reverse transcription in the absence of an exogenous primer. cDNA was diluted 1/50 and 1/100, and replicate reactions were run for each dilution. The vRNA used was extracted from the plasma of participant 25. M; molecular weight marker.

**Table S1** Characteristics of study participants.

| Participant ID | Sample date | Viral load (log_10_ copies/ml) | CD4 (cells/mm^3^) | Infection stage | Treatment |
| --- | --- | --- | --- | --- | --- |
| 09 | 18/02/2022 | 6,67 | NA | Acute | Naive |
| 25 | 11/06/2021 | 7,30 | 360 | Acute | Naive |
| 30 | 30/06/2021 | 6,90 | 272 | Acute | Naive |
| 32 | 25/07/2022 | 3,78 | 237 | Chronic | ART |
| 34 | 12/07/2022 | 4,14 | 538 | Chronic | Naive |
| 42 | 24/10/2022 | 4,70 | 136 | Chronic | ART |
| 63 | 18/11/2020 | 5,80 | 487 | Acute | Naive |
| 82 | 01/02/2022 | 4,96 | 216 | Chronic | ART interruption |

**Table S2** Raw data (Cq values) of the LTR and INT qPCR reactions. The vRNA used was extracted from plasma of participants 30, 09 and 63. Reverse transcription was performed with different reverse transcriptases: SuperScript IV (SSIV), PrimeScript II (PSII), Transcriptor (TRT) and MMuLV. Reactions were initiated with random hexamers (RH6), anchored oligodT 20 (odT20) or in the absence of an exogenous primer (NP). All qPCRs were performed in triplicate. ND; not exceeding the threshold.

| Participant | qPCR | Primer | SSIV | | | PSII | | | TRT | | | MMuLV | | |
| --- | --- | --- | --- | --- | --- | --- | --- | --- | --- | --- | --- | --- | --- | --- |
| 30 | LTR | RH6 | 23.92 | 24.11 | 23.74 | 22.69 | 22.69 | 22.53 | 22.55 | 22.73 | 22.58 | 24.25 | 24.25 | 24.20 |
|  |  | odT20 | 23.04 | 22.40 | 22.39 | 22.88 | 22.89 | 22.73 | 22.90 | 23.13 | 22.82 | 23.59 | 23.69 | 23.83 |
|  |  | NP | 23.23 | 23.48 | 23.53 | 22.96 | 23.00 | 22.96 | 22.88 | 22.85 | 22.82 | 25.98 | 26.00 | 25.82 |
|  | INT | RH6 | 23.68 | 24.34 | 24.43 | 23.68 | 23.65 | 24.05 | 23.73 | 23.74 | 23.68 | 22.91 | 22.88 | 22.90 |
|  |  | odT20 | 23.69 | 23.50 | 23.56 | 25.72 | 26.52 | 25.80 | 25.58 | 25.66 | 25.46 | 24.45 | 24.51 | 24.50 |
|  |  | NP | 29.56 | 28.99 | 28.90 | 32.61 | 33.90 | 33.70 | 33.32 | 32.74 | 33.14 | 33.00 | 31.79 | 32.10 |
| 09 | LTR | RH6 | 24.82 | 24.82 | 24.87 | 24.88 | 24.68 | 24.77 | 25.83 | 25.08 | 25.46 | 26.61 | 26.76 | 26.66 |
|  |  | odT20 | 24.96 | 25.09 | 25.01 | 25.14 | 25.05 | 25.12 | 25.63 | 25.55 | 25.56 | 25.60 | 25.71 | 25.82 |
|  |  | NP | 26.06 | 25.73 | 25.91 | 25.80 | 25.74 | 25.73 | 25.86 | 25.83 | 26.24 | 28.08 | 28.37 | 28.49 |
|  | INT | RH6 | 25.34 | 25.28 | 25.25 | 25.39 | 25.47 | 25.50 | 26.31 | 26.68 | 26.70 | 26.27 | 26.15 | 26.20 |
|  |  | odT20 | 26.14 | 26.03 | 26.10 | 28.94 | 29.18 | 29.47 | 27.26 | 27.49 | 27.30 | 26.69 | 26.70 | 26.76 |
|  |  | NP | 31.28 | 32.02 | 31.50 | 34.07 | 34.59 | 34.13 | 34.11 | 35.90 | 34.53 | 35.24 | 34.53 | 33.29 |
| 63 | LTR | RH6 | 26.52 | 26.90 | 26.75 | 27.10 | 27.00 | 27.17 | 27.16 | 27.50 | 27.34 | 28.19 | 28.61 | 28.41 |
|  |  | odT20 | 27.63 | 27.16 | 26.85 | 27.63 | 27.78 | 27.86 | 27.30 | 26.88 | 26.99 | 28.55 | 28.66 | 28.23 |
|  |  | NP | 28.08 | 27.98 | 27.97 | 28.05 | 27.87 | 27.95 | 28.19 | 28.17 | 27.99 | 31.56 | 31.13 | 31.78 |
|  | INT | RH6 | 26.43 | 26.43 | 26.62 | 27.54 | 27.67 | 27.72 | 28.64 | 28.45 | 28.51 | 27.64 | 27.71 | 27.61 |
|  |  | odT20 | 27.83 | 27.85 | 27.71 | 29.24 | 29.10 | 29.24 | 29.21 | 29.25 | 29.18 | 28.25 | 28.60 | 28.51 |
|  |  | NP | 32.95 | 32.77 | 32.72 | 35.09 | 35.02 | 35.30 | ND | ND | 35.64 | 35.76 | 35.05 | ND |

**Table S3** Raw data (Cq values) of the LTR qPCR reactions on cDNA obtained from RNA pretreated at different conditions: heating at 65°C or 95°C, with and without the addition of a competitor primer (C). The vRNA used was extracted from plasma of participants 30 and 09. Reverse transcription was performed with SuperScript IV (SSIV) and Transcriptor (TRT) and using random hexamers (RH6) to initiate the reaction or in absence of an exogenous primer (NP). All qPCRs were performed in triplicate. NE; no enzyme control reactions, C; competitor primer, NA; not applicable.

| Participant | Enzyme | Primer | Condition | LTR | | |
| --- | --- | --- | --- | --- | --- | --- |
| 30 | SSIV | RH6 | 65°C | 22.66 | 22.69 | 22.71 |
|  |  |  | 95°C | 23.09 | 23.12 | 22.97 |
|  |  | NP | 65°C | 23.50 | 23.45 | 23.46 |
|  |  |  | 95°C | 28.76 | 28.69 | 28.93 |
|  |  |  | 95°C+C | 29.73 | 29.69 | 29.54 |
|  | TRT | RH6 | 65°C | 23.48 | 23.46 | 23.24 |
|  |  |  | 95°C | 24.35 | 24.49 | 24.16 |
|  |  | NP | 65°C | 24.56 | 24.12 | 24.16 |
|  |  |  | 95°C | 31.31 | 30.62 | 31.45 |
|  |  |  | 95°C+C | 31.48 | 31.57 | 32.05 |
|  |  | NE | 65°C | 0.00 | NA | NA |
|  |  |  | 95°C | 0.00 | NA | NA |
|  |  |  | 95°C+C | 0.00 | NA | NA |
| 09 | SSIV | RH6 | 65°C | 24.89 | 25.10 | 25.12 |
|  |  |  | 95°C | 25.05 | 24.97 | 25.09 |
|  |  | NP | 65°C | 26.01 | 25.94 | 25.76 |
|  |  |  | 95°C | 30.66 | 30.52 | 30.80 |
|  |  |  | 95°C+C | 31.51 | 31.06 | 31.17 |
|  | TRT | RH6 | 65°C | 26.54 | 26.30 | 26.40 |
|  |  |  | 95°C | 27.87 | 27.64 | 27.69 |
|  |  | NP | 65°C | 25.03 | 25.61 | 25.70 |
|  |  |  | 95°C | 33.82 | 32.84 | 33.38 |
|  |  |  | 95°C+C | 33.55 | 33.65 | 34.27 |
|  |  | NE | 65°C | 0.00 | NA | NA |
|  |  |  | 95°C | 0.00 | NA | NA |
|  |  |  | 95°C+C | 0.00 | NA | NA |

**Table S4** Details and sequences of the primers and probes used for reverse transcription, qPCR, nested PCR, and sequencing.

| Primer name | Position relative to HXB2 | Orientation | Sequence (5' → 3') | Function |
| --- | --- | --- | --- | --- |
| LTR_REV | 622 → 642 | antisense | GGCGCCACTGCTAGAGATTTT | LTR qPCR |
| LTR_FW | 522 ← 539 | sense | GCCTCAATAAAGCTTGCC | LTR qPCR |
| LTR_PROBE | 551 → 565 | sense | FAM-AAGTRGTGTGTGCCC-BHQ1 | LTR qPCR |
| INT_FW | 4901 → 4924 | sense | GGTTTATTACAGGGACAGCAGAGA | INT qPCR |
| INT_REV | 5040 ← 5060 | antisense | ACCTGCCATCTGTTTTCCATA | INT qPCR |
| INT_PROBE | 4953 ← 4978 | antisense | FAM-ACTACTGCCCCTTCACCTTTCCARAG-TAMRA | INT qPCR |
| F1fw1 | 524 → 547 | sense | CTCAATAAAGCTTGCCTTGAGTGC | LTR PCR + Primer in RT2 for tRNA(Lys) RT + tRNA PCR |
| F1fw2 | 551 → 571 | sense | AAGTAGTGTGTGCCCGTCTGT | LTR PCR + tRNA PCR |
| 633AS+P7 | 606 ← 633 | antisense | CAAGCAGAAGACGGCATACGAGATGCTAGAGATTTTCCACACTGACTAAAAG | LTR PCR + U3-U5 PCR +Gag-U5 PCR |
| P7 | NA | antisense | CAAGCAGAAGACGGCATACGAGAT | LTR PCR + tRNA PCR + U3-U5 PCR + Gag-U5 PCR |
| 4235 | 4653 → 4675 | sense | CCCTACAATCCCCAAAGTCAAGG | INT PCR |
| 4327 | 4745 → 4767 | sense | TAAGACAGCAGTACAAATGGCAG | INT PCR |
| 4538 | 4956 ← 4976 | antisense | TACTGCCCCTTCACCTTTCCA | INT PCR |
| 4481 | 4899 ← 4919 | antisense | GCTGTCCCTGTAATAAACCCG | INT PCR |
| PBS | 636 → 653 | sense | TGGCGCCCGAACAGGGAC | Competitor primer |
| tRNA+P7 | NA | NA | CAAGCAGAAGACGGCATACGAGATGGATAGCTCAGTCGGTAGAGCA | tRNA PCR |
| LTR72 | 72 → 96 | sense | TTCCCTGATTRGCAGAACTACACAC | U3-U5 PCR |
| LTR143 | 140 → 162 | sense | CAAGCTAGTACCAGTTGAGCCAG | U3-U5 PCR |
| DNAF2 | 682 →705 | sense | TCTCTCGACGCAGGACTCGGCTTG | Gag-U5 PCR + Gag-U3 PCR |
| 708S | 708 →724 | sense | GAAGCGCGCWCGGCAAG | Gag-U5 PCR + Gag-U3 PCR |
| NefREV3 | 9171 ← 9190 | antisense | CTGGCCCTGGTGTGTAGTTC | Gag-U3 PCR |
| Nefyn05 | 9157 ← 9181 | antisense | GTGTGTAGTTCTGCCAATCAGGGAA | Gag-U3 PCR |
| 1231F | 1231 →1255 | sense | TCACCTAGAACTTTRAATGCATGGG | Sanger sequencing |
| 1817F | 1817 → 1834 | sense | TAGAAGAAATGATGACAG | Sanger sequencing |
| F2fw2 | 2022 → 2039 | sense | GGGCTGTTGGARATGTGG | Sanger sequencing |
| F1rev2 | 2252 ← 2272 | antisense | TGCCAAAGAGTGATYTGAGGG | Sanger sequencing |
| RT2955 | 2955 ← 2977 | antisense | CTAATYCCTGGYGTYTCATTRTT | Sanger sequencing |
| INMW1 | 2997 → 3019 | sense | CCACARGGATGGAAAGGATCACC | Sanger sequencing |
| R5 | 3492 ← 3511 | antisense | GGGTCATAATACACTCCATG | Sanger sequencing |
| F3fw2 | 3681 → 3703 | sense | GAAAGCATAGTRATATGGGGAAA | Sanger sequencing |
| F2rev2 | 3777 ← 3799 | antisense | ACAAACTCCCAYTCAGGAATCCA | Sanger sequencing |
| IN99 | 4504 ← 4482 | antisense | TCTGCTGGRATRACYTCTGCYTC | Sanger sequencing |
| F3rev2 | 5040 → 5061 | antisense | CACCTGCCATCTGTTTTCCATA | Sanger sequencing |
| F4fw2 | 4956 → 4974 | sense | TGGAAAGGTGAAGGGGCAG | Sanger sequencing |
| EnvoutF1 | 5550 → 5574 | sense | AGARGAYAGATGGAACAAGCCCCAG | Sanger sequencing |
| 5861F | 5861 →5884 | sense | TGGAAGCATCCRGGAAGTCAGCCT | Sanger sequencing |
| F4rev2 | 6429 ←6450 | antisense | GTACACAGGCATGTGTRGCCCA | Sanger sequencing |
| 6951 | 6951 →6973 | sense | AGYRCAGTACAATGYACACATGG | Sanger sequencing |
| 7336 | 7318 ← 7336 | antisense | ATTTCTGGRTCYCCKCCTG | Sanger sequencing |
| 7238 | 7645 ← 7667 | antisense | ACTTCTCCAATTGTCCCTCATAT | Sanger sequencing |
| 8445R | 8424 ← 8445 | antisense | CTCTCTCTCCACCTTCTTCTTC | Sanger sequencing |
